# Lower birth weight is linked to poorer cardiovascular health in middle-aged population-based adults

**DOI:** 10.1136/heartjnl-2022-321733

**Published:** 2022-11-16

**Authors:** Zahra Raisi-Estabragh, Jackie Cooper, Mae S Bethell, Celeste McCracken, Adam J Lewandowski, Paul Leeson, Stefan Neubauer, Nicholas C Harvey, Steffen E Petersen

**Affiliations:** 1 Barts Heart Centre, Saint Bartholomew's Hospital, Barts Health NHS Trust, London, UK; 2 William Harvey Research Institute, NIHR Barts Biomedical Research Centre, Queen Mary University of London, London, UK; 3 Imperial College Healthcare NHS Trust, London, UK; 4 Division of Cardiovascular Medicine, Radcliffe Department of Medicine, University of Oxford, National Institute for Health Research Oxford Biomedical Research Centre, Oxford University Hospitals NHS Foundation Trust, Oxford, UK; 5 MRC Lifecourse Epidemiology Centre, Southampton, UK; 6 NIHR Southampton Biomedical Research Centre, Southampton, UK; 7 Health Data Research UK, London, UK; 8 Alan Turing Institute, London, UK

**Keywords:** Epidemiology, Risk Factors, Magnetic Resonance Imaging

## Abstract

**Objective:**

To examine associations of birth weight with clinical and imaging indicators of cardiovascular health and evaluate mechanistic pathways in the UK Biobank.

**Methods:**

Competing risk regression was used to estimate associations of birth weight with incident myocardial infarction (MI) and mortality (all-cause, cardiovascular disease, ischaemic heart disease, MI), over 7–12 years of longitudinal follow-up, adjusting for age, sex, deprivation, maternal smoking/hypertension and maternal/paternal diabetes. Mediation analysis was used to evaluate the role of childhood growth, adulthood obesity, cardiometabolic diseases and blood biomarkers in mediating the birth weight–MI relationship. Linear regression was used to estimate associations of birth weight with left ventricular (LV) mass-to-volume ratio, LV stroke volume, global longitudinal strain, LV global function index and left atrial ejection fraction.

**Results:**

258 787 participants from white ethnicities (61% women, median age 56 (49, 62) years) were studied. Birth weight had a non-linear relationship with incident MI, with a significant inverse association below an optimal threshold of 3.2 kg (subdistribution HR: 1.15 (1.08 to 1.22), p=6.0×10^–5^) and attenuation to the null above this threshold. The birth weight–MI effect was mediated through hypertension (8.4%), glycated haemoglobin (7.0%), C reactive protein (6.4%), high-density lipoprotein (5.2%) and high cholesterol (4.1%). Birth weight–mortality associations were statistically non-significant after Bonferroni correction. In participants with cardiovascular magnetic resonance (n=19 314), lower birth weight was associated with adverse LV remodelling (greater concentricity, poorer function).

**Conclusions:**

Lower birth weight was associated with greater risk of incident MI and unhealthy LV phenotypes; effects were partially mediated through cardiometabolic disease and systemic inflammation. These findings support consideration of birth weight in risk prediction and highlight actionable areas for disease prevention.

**WHAT IS ALREADY KNOWN ON THIS TOPIC**

- Previous studies have linked prematurity and small-for-gestational age (SGA) with increased lifetime cardiovascular risk. It is unclear whether similar risks are associated with low birth weight outside of these specific contexts.

**WHAT THIS STUDY ADDS**

- We demonstrate association of lower birth weight with higher risk of incident myocardial infarction and adverse left ventricular remodelling in a large population-based cohort of middle-aged adults. Associations were partially mediated through cardiometabolic diseases and systemic inflammation.

**HOW THIS STUDY MIGHT AFFECT RESEARCH, PRACTICE OR POLICY**

- Our findings indicate that the adverse cardiovascular effects of low birth weight described in preterm/SGA cohorts may extend significantly outside of these contexts. Further research is required to determine whether inclusion of birth weight may improve existing risk stratification tools and if preventative strategies targeted at individuals with lower birth weight have a role in improving clinical outcomes.

## Introduction

The fetal origins hypothesis proposes intrauterine undernutrition as a key driver of increased susceptibility to later ischaemic heart disease (IHD) and associated mortality.^1^ Many epidemiological studies have demonstrated association of low birth weight with prevalent and incident IHD, and with mortality outcomes.^1–5^ Others, however, have questioned the public health importance of the relationship.^6^ Many older studies do not account for key potential confounding or mediating factors, with more recent evidence suggesting that the range of cardiovascular outcomes convincingly associated with birth weight is more limited than originally described.^7^ Importantly, it is not clear whether the adverse cardiovascular impact of low birth weight described in the settings of preterm birth or small-for-gestational age (SGA) extends to a wider population effect outside of these specific contexts.

While several studies have reported the association between low birth weight and a range of intermediary cardiometabolic factors, including insulin resistance,^5^ type 2 diabetes (T2D)^8^ and high blood pressure,^9^ the formal role of these conditions in mediating the relationships between birth weight and clinical cardiovascular outcomes has not been examined.

Cardiovascular imaging phenotypes reflect organ-level remodelling in response to a wide range of exposures and provide reliable indicators of cardiovascular health. The links between birth weight and cardiovascular image-derived phenotypes in large population-based cohorts have not been previously reported. Such analyses may provide new insights into the cardiovascular impact of birth weight, allowing capture of effects both independent of, and acting through, potential cardiometabolic mediators (eg, hypertension, diabetes).

We examined the association of birth weight with disease-specific mortality outcomes and incident acute myocardial infarction (AMI) in 258 787 UK Biobank participants. We accounted for potential confounders and evaluated the mediating role of childhood growth, cardiometabolic diseases, and blood markers of lipid profile, glycaemic control, and systemic inflammation. Finally, we assessed the association of birth weight with cardiovascular magnetic resonance (CMR) measures of cardiovascular structure and function.

## Methods

### Setting and study population

The UK Biobank comprises half a million participants recruited aged 40–69 years between 2006 and 2010. The research protocol is publicly available.^10^ Health record linkage permits prospective tracking of health events for all participants. Adjudicated algorithmically defined outcomes are produced for key illnesses. The UK Biobank Imaging Study includes CMR and aims to scan 20% of the original participants (2015–ongoing).

### Ascertainment of birth weight

Birth weight was self-reported at baseline assessment. We included singleton births with birth weights between 1 and 5 kg. Given known ethnic variations in birth weight,^11^ we limited to white ethnicities, which comprise >97% of the cohort.

### Ascertainment of outcomes

The following mortality outcomes were extracted from death register records documented per International Classification of Disease codes: any cardiovascular disease (CVD) (IX), IHD (I20–I25). Fatal AMI comprised of cases with AMI as the primary cause of death. Incident AMI was obtained from algorithmically defined outcome data; participants with a record of AMI at baseline were excluded from the analysis for this outcome. Outcomes occurring from baseline to the latest available censor dates were included (mortality: 31 January 2018, AMI: 31 March 2017) giving 7–12 years of follow-up.

### Cardiovascular structure and function metrics

CMR was performed according to a predefined protocol and analysed using an automated pipeline.^12^ The following measures were included: left ventricular (LV) mass to LV end-diastolic volume ratio (LVM/LVEDV), LV stroke volume (LVSV), global longitudinal strain (GLS), LV global function index (LVGFI), left atrial ejection fraction (LAEF).

### Statistical analysis

Statistical analysis was performed using R V.4.1.0 (R Core Team (2022). R: A language and environment for statistical computing. R Foundation for Statistical Computing, Vienna, Austria. https://www.R-project.org/) and Stata V.17 (StataCorp. 2021. Stata Statistical Software: Release 17. College Station, Texas, USA: StataCorp). Descriptive statistics are presented as mean (SD) or median (25th percentile, 75th percentile) depending on skew for continuous variables and number (percentage) for categorical data. We first estimated the association of birth weight with the incident outcomes. All-cause mortality models were fitted using Cox regression. Other outcomes were analysed using competing risk regression with proportional subdistribution hazards fitted as per Fine and Gray. Any mortality outcome occurring in patients without the outcome of interest was treated as a competing event. Sensitivity analysis was conducted using cause-specific Cox regression. Models were adjusted for age, sex, deprivation, maternal smoking, maternal diabetes, maternal hypertension and paternal diabetes (online supplemental table 1). We report HRs for all-cause mortality, and subdistribution HRs (SHRs) for all other outcomes, with corresponding 95% CIs per 1 kg increase in birth weight. Plots of martingale residuals were used to check functional form. A key assumption of survival models is a linear association between log-hazard and continuous covariates, and failure to assess non-linearity may result in loss of information and incorrect interpretation of results. We therefore characterised possible non-linearity using restricted cubic spline models, which provide a flexible way to model more complex relationships between birth weight and outcome. The number of knots was selected by model fit and optimal cut-offs were identified using the Youden index. Statistically significant results are thus also presented in categories above and below the identified birth weight cut-off.

10.1136/heartjnl-2022-321733.supp1 Supplementary data

We selected potential biological mediators of the birth weight–outcome relationships based on knowledge of potential causal pathways. A directed acyclic graph sets out the causal framework assumed by our models (online supplemental figure 1). The following mediating variables were included: diabetes, hypercholesterolaemia, obesity, hypertension, comparative height/weight aged 10 years old, glycated haemoglobin (HbA1c), glucose, low-density lipoprotein, high-density lipoprotein (HDL), triglyceride, apolipoprotein A and C reactive protein (CRP). Mediation analysis was performed based on an accelerated failure time model as per Fulcher *et al* 13 using a regression-based approach, which extended to allow for multiple mediators.^14^ Two models were fitted: (1) a model for outcome including all mediators, covariates (age, sex, deprivation, maternal smoking, maternal diabetes, maternal hypertension, paternal diabetes), and birth weight as predictors; (2) models for each mediator including covariates and birth weight. No significant exposure–mediator interactions were observed. The direct effect was given by the coefficient for birth weight in the outcome model. The indirect effect was calculated by the product of the coefficient for birth weight in the mediator model and the coefficient for the mediator in the outcome model. For binary mediators, a logistic mediator model was fitted, and the expression for the indirect effect was modified to ensure appropriate scaling^14^ CIs were constructed using bootstrap resampling. We thus calculated the indirect effect of each mediator, the overall direct and total effect. We present the proportion of effect independently mediated by each variable as a percentage of the total effect. Sensitivity analysis was conducted to assess the impact of missing data. Imputation was conducted using stochastic regression imputation in the MICE package in R, and the mediation analysis was repeated.

We estimated the association of birth weight with CMR metrics using linear regression, adjusting for confounders as before. We report change in CMR measure per 1 kg increase in birth weight with corresponding 95% CIs and p values. We used Bonferroni correction for multiple testing based on number of outcomes with significance thresholds indicated in table footnotes.

## Results

### Baseline characteristics

The analysis sample comprised 258 787 individuals (100 540 men and 158 247 women) with median age of 56 (49, 62) years (online supplemental figure 2). The mean birth weight was 3.34 (±0.60) kg. The rates of cardiac risk factors were as follows: diabetes 4.2%, hypertension 25.0%, hypercholesterolaemia 15.6%, smoking 9.8%. CMR was available for 19 314 participants; this subset had slightly healthier cardiometabolic profile than the baseline cohort (table 1).

**Table 1:** Baseline participants’ characteristics

	Baseline cohort (n=258 787)	CMR subset (n=19 314)
Age	56 (49, 62)	62 (56, 68)
Men	100 540 (38.9%)	8074 (41.8%)
Birth weight (kg)	3.34 (±0.60)	3.37 (±0.57)
Diabetes	10 760 (4.2%)	445 (2.3%)
Hypertension	64 724 (25.0%)	3489 (18.1%)
Hypercholesterolaemia	40 297 (15.6%)	2141 (11.1%)
BMI (kg/m^2^)	27.3 (±4.9)	25.9 (23.5, 28.8)
Smoking (current)	25 388 (9.8%)	1154 (6.0%)
Townsend deprivation score	−2.33 (−3.74, 0.10)	−2.69 (−3.9, 0.71)
Maternal smoking	68 654 (29.6%)	5193 (26.9%)
Paternal diabetes	23 566 (9.1%)	1882 (9.7%)
Maternal diabetes	23 889 (9.2%)	1788 (9.3%)
Maternal hypertension	85 355 (33.0%)	6577 (34.1%)
LVM/LVEDV (g/mL)	–	0.58 (±0.09)
LVSVi (mL/m^2^)	–	46.9 (±8.2)
LVGFI (%)	–	0.48 (±0.07)
GLS (%)	–	−18.6 (±2.7)
LAEF (%)	–	61.6 (±8.8)

BMI, body mass index; CMR, cardiovascular magnetic resonance; GLS, global longitudinal strain; LAEF, left atrial ejection fraction; LVGFI, left ventricular global function index; LVM/LVEDV, left ventricular mass to left ventricular end-diastolic volume ratio; LVSVi, left ventricular stroke volume index.

Duration of follow-up from baseline to the censor dates gave total person time of 2 304 749 years (median 9.0 (8.3, 9.7) years) for mortality outcomes, and 2 069 086 years (median 8.2 (7.5, 8.8) years) for incident AMI. The absolute number of outcomes and the rate of outcomes per 1000 person-years are presented in online supplemental table 2.

### Association of birth weight with incident outcomes

**Figure 1:**
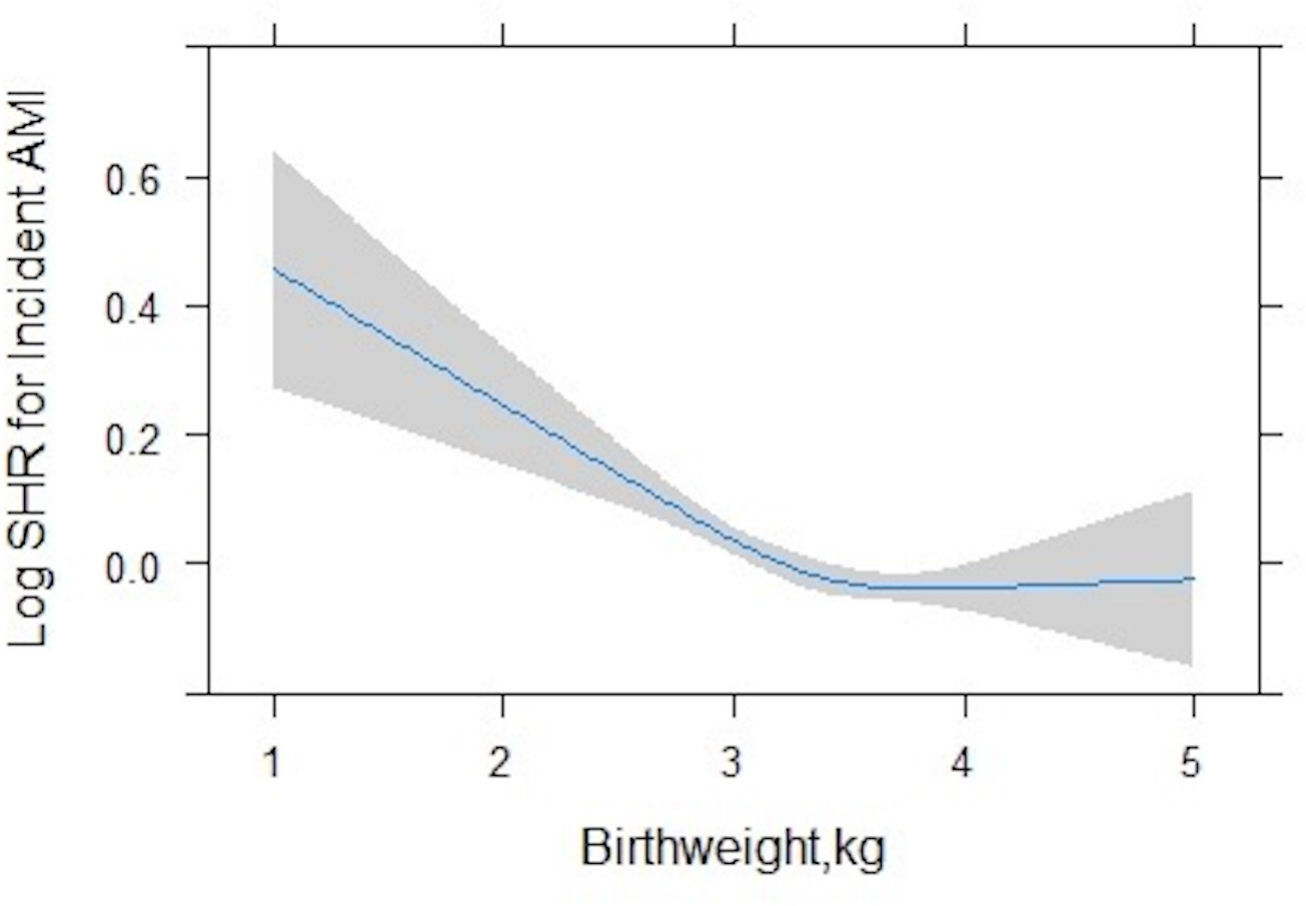
Three-knot cubic spline model demonstrating relationship between birth weight and hazard of incident acute myocardial infarction (AMI) fully adjusted models. Blue line gives estimated curve with 95% CI shown by grey-shaded area. SHR, subdistribution HR.

In fully adjusted models testing the linear association, higher birth weight was associated with reduction in hazard of all outcomes tested. After multiple testing correction, only association with incident MI remained statistically significant (table 2). There was evidence of significant non-linearity in birth weight–AMI association (online supplemental table 3). The best model fit for this relationship was a cubic spline model with three knots (online supplemental table 4). Plotting this model (fully adjusted) revealed a non-linear relationship with a significant inverse association below an optimal threshold of 3.2 kg and attenuation to the null above this threshold (figure 1). Among individuals with birth weight less than 3.2 kg, each 1 kg increase in birth weight was associated with approximately 15% reduction in hazard of incident AMI (SHR 0.85 (0.76 to 0.95), p=0.005) (table 3). Sensitivity analysis using cause-specific Cox regression gave near identical results.

**Table 2:** Association of birth weight with incident outcomes (baseline cohort)

	Univariate	Age and sex adjusted	Fully adjusted
All-cause mortality	1.02 (0.98 to 1.05) p=0.41	0.97 (0.94 to 1.00) p=0.073	0.98 (0.95 to 1.01) p=0.210
CVD death	1.01 (0.92 to 1.10) p=0.88	0.90 (0.83 to 0.97) p=0.010	0.91 (0.84 to 0.99) p=0.028
IHD death	1.02 (0.91 to 1.16) p=0.70	0.86 (0.77 to 0.96) p=0.009	0.88 (0.79 to 0.98) p=0.020
Fatal AMI	1.04 (0.86 to 1.26) p=0.67	0.89 (0.75 to 1.05) p=0.16	0.90 (0.76 to 1.06) p=0.216
Incident AMI	1.00 (0.94 to 1.06) p=0.90	0.89 (0.84 to 0.93) p=1.0×10^−5^	0.90 (0.85 to 0.95) p=7.0×10^–5^

All cause-mortality associations are estimated using Cox proportional hazard models, results are expressed as HR (95% CI) and p value. For all other outcomes, competing risk regression models are used with results expressed as subdistribution HRs (95% CI) and p values. For all outcomes, results are per 1 kg increase in birth weight. Fully adjusted models include adjustment for age, sex, social deprivation, maternal diabetes, maternal hypertension, maternal smoking and paternal diabetes. Statistical significance level is p<0.01, corrected for five outcomes.

AMI, acute myocardial infarction; CVD, cardiovascular disease; IHD, ischaemic heart disease.

**Table 3:** Subdistribution HRs for the within-group and between-group effect of birth weight on incident acute myocardial infarction (AMI)

Outcomes	Birth weight <3.2 kg n=104 890 (1554 events)	Birth weight ≥3.2 kg n=149 280 (2083 events)	P value between group difference
Incident AMI, within-group effects	0.85 (0.76 to 0.95) p=0.005	1.01 (0.91 to 1.13) p=0.80	p=0.02
Incident AMI, between-group effects	1.15 (1.08 to 1.22)	1.00	p=6.0×10^−5^

Models adjusted for age, sex, social deprivation, maternal diabetes, maternal hypertension, maternal smoking and paternal diabetes. Results are subdistribution HRs per 1 kg increase in birth weight with corresponding 95% CIs. Significance level is set at p<0.025 for within-group and p<0.05 for between-group effects.

### Mediation analysis

**Figure 2:**
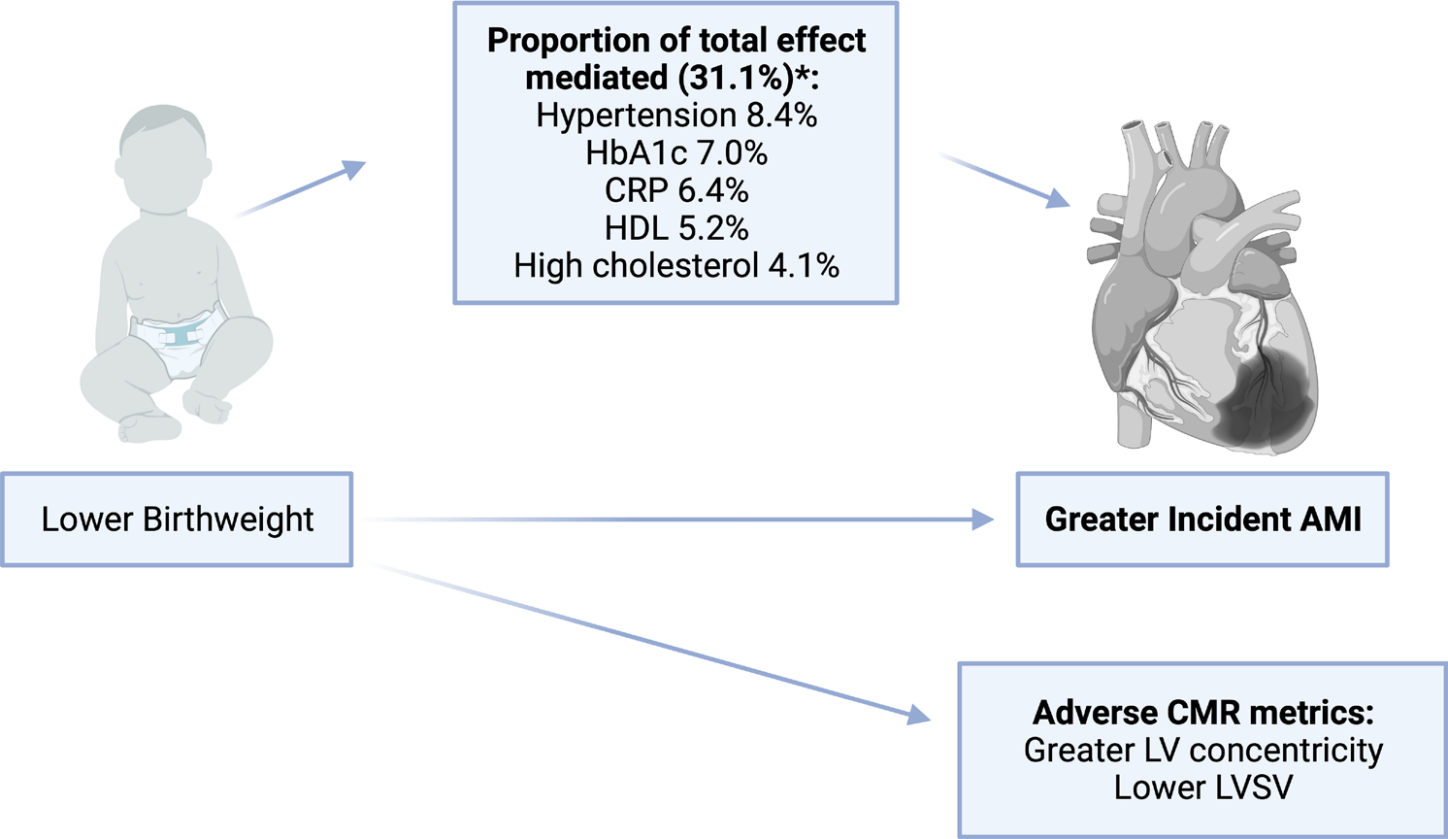
Summary of the adverse cardiovascular imaging and clinical associations of birth weight and key mediating variables. *Includes significant independent mediator variables. AMI, acute myocardial infarction; CMR, cardiovascular magnetic resonance; CRP, C reactive protein; HbA1c, glycated haemoglobin; HDL, high-density lipoprotein; LV, left ventricle; LVSV, left ventricular stroke volume.

We considered mediation of the birth weight–AMI relationship. Multiple mediation analysis was used to calculate independent indirect effects for each mediator; this represents the effect of birth weight on incident AMI acting through individual mediators expressed as HRs and also, for ease of interpretation, as percentages (online supplemental table 5). We detected significant mediating effect through hypertension (8.4%), HbA1c (7.0%), CRP (6.4%), HDL (5.2%) and high cholesterol (4.1%). The proportion of the total effect mediated through all statistically significant mediators was 31.1% (figure 2). Sensitivity analysis with imputed mediator variables showed similar results (online supplemental table 6).

### Association of birth weight with CMR indices

In fully adjusted linear regression models, lower birth weight was associated with more concentric pattern of LV remodelling (higher LVM/LVEDV) and poorer LV function (lower LVSV index). Associations with LVGFI, GLS and LAEF were not statistically significant in fully adjusted models (table 4).

**Table 4:** Associations of birth weight with CMR measures of cardiovascular structure and function

	Unadjusted	Age and sex adjusted	Fully adjusted
	β (95% CI)	β (95% CI)	β (95% CI)
LVM/LVEDV (g/mL)	0.005 (0.002 to 0.007) p=3.3×10^–5^	−0.004 (–0.006 to 0.002) p=0.0003	−0.004 (–0.006 to 0.002) p=0.0005*
LVSVi (mL/m^2^)	1.16 (0.96 to 1.36) p=2.65×10^–29^	0.70 (0.50 to 0.90) 4.18×10^–12^	0.70 (0.51 to 0.90) 3.96×10^–12^*
LVGFI (%)	−0.007 (–0.008 to 0.005) p=1.29×10^–14^	0.001 (–0.001 to 0.002) p=0.31	0.001 (–0.001 to 0.002) p=0.30
GLS (%)	0.11 (0.03 to 0.18) p=0.005	−0.07 (–0.14 to 0.01) p=0.08	−0.07 (–0.14 to 0.01) p=0.07
LAEF (%)	0.11 (–0.13 to 0.35) p=0.36	0.27 (0.03 to 0.51) p=0.03	0.25 (0.01 to 0.49) p=0.04

Results are from linear regression models with CMR metrics set as model outcome (response variable) and birth weight set as the exposure of interest; covariate adjustment is as indicated in column heads; fully adjusted models include age, sex, social deprivation, maternal diabetes, maternal hypertension, maternal smoking and paternal diabetes. Reported betas are change in CMR metric per 1 kg increase in birth weight. Statistical significance level is p<0.01, corrected for five outcomes.

*Statistically significant results for fully adjusted results.

CMR, cardiovascular magnetic resonance; GLS, global longitudinal strain; LAEF, left atrial ejection fraction; LVGFI, left ventricular global function index; LVM/LVEDV, left ventricular mass to left ventricular end-diastolic volume ratio; LVSVi, left ventricular stroke volume index.

## Discussion

### Summary of findings

In this population-based study of 258 787 middle-aged and older-aged adults, birth weight had a non-linear relationship with incident AMI, with a significant inverse association below an optimal threshold of 3.2 kg and attenuation to the null above this threshold. Mediation analysis identified five independent mediators of the birth weight–AMI relationship, which together explained around one-third of the total effect. These comprised indicators of poor glycaemic control (HbA1c 7.0%), adverse lipid profile (HDL 5.2%, high cholesterol 4.1%), hypertension (8.4%) and systemic inflammation (CRP 6.4%). Among 19 314 participants with CMR performed as part of the UK Biobank Imaging Study, lower birth weight was linked to adverse LV structure (higher LVM/LVEDV) and function (lower LVSV).

### Associations with incident events

Inverse associations between birth weight and prevalent and incident IHD, and IHD mortality have been widely reported. In a study of 1394 post-menopausal women, Lawlor *et al* 5 report a significant association between low birth weight and prevalent IHD, as identified from primary care records. In a large national Swedish cohort of 1.98 million young adults, Zoller *et al* 3 report significant association of low birth weight with incident IHD, defined as secondary or primary diagnosis on hospitalisation records. This association appeared stronger when the disease outcome was set more specifically to AMI.^3^ These relationships appeared independent of socioeconomic status, cardiometabolic disease and pregnancy-related factors. A study of 10 803 individuals from Scotland^2^ reports significant association of lower birth weight with greater risk of incident IHD, defined as a composite of IHD hospitalisation and deaths. As in our study, the authors report no modifying effect of childhood height or weight on the effect of birth weight.^2^ However, the authors were unable to consider the potential mediating role of cardiometabolic factors. In a large population-based cohort study of 15 000 individuals, Leon *et al* 4 report inverse association of low birth weight with IHD death; however, several key confounders were overlooked in this study.

Our analysis demonstrates a convincing significant inverse relationship between birth weight and incident AMI. Characterisation of the non-linearity of the birth weight–AMI relationship in our study demonstrated a negative linear association for birth weights below 3.2 kg and no clear association with risk for birth weights above this threshold. The cut-off identified by our model approximates the population average birth weight,^15^ suggesting that the adverse cardiovascular effects observed are not restricted to low birthweight (<2.5 kg) individuals,^16^ but rather impacting a much larger proportion of the population. We observed significant associations of birth weight with IHD death and CVD death, but these relationships were statistically non-significant after application of a Bonferroni correction. It is possible that previous reports of birth weight–mortality associations may be influenced by residual confounding. The other consideration is possible lack of power in our study. In another analysis from the UK Biobank, as with our findings, Liang *et al* 17 report non-linear inverse association of birth weight with incident IHD. Our results reproduce these findings with a more disease-specific outcome (AMI), and extend the work with inclusion of mortality outcomes, consideration of mechanisms through mediation analysis and evaluation of relationships with CMR phenotypes.

### Biological mechanisms

Previous studies have linked low birth weight to adverse cardiometabolic disease. In a study of 72 healthy children, Hofman *et al* 18 report reduction in insulin sensitivity, a risk factor for type 2 diabetes (T2D), in children who were born at term but were SGA. Indeed, in a large meta-analysis of 135 studies, Knop *et al* 19 conclude that birth weight is linked to T2D in a J-shaped manner, somewhat mirroring the characterisation of the birth weight–AMI association in our study. In an analysis of the Nurses’ Health Study and the Health Professionals Follow-Up Study, Wang *et al* 8 report significant association of genetically lowered birth weight with increased susceptibility to T2D. In a Mendelian randomisation study of the UK Biobank, Zanetti *et al* 20 similarly report a causal relationship between genetically determined lower birth weight and T2D. Our findings, using multiple mediation analysis, identified serum HbA1c as one of the most important mediators in the birth weight–AMI relationship. Our study corroborates existing evidence using a novel approach and adds new information by formally demonstrating and quantifying the mediating role of glycaemic control in driving associations of birth weight with incident AMI.

Several large cohort studies from the UK^9^ and the USA^21^ have linked lower birth weight with greater likelihood of hypertension in adulthood. Consistently, we identified hypertension as an important mediator of the birth weight–AMI relationship. We observed a more modest role for high cholesterol and serum HDL, which is consistent with previous observations of the association of lower birth weight with adverse serum lipids.^22^

We identified a significant mediating effect from higher CRP levels. Previous work has demonstrated inverse association of birth weight and serum CRP in both children^23^ and adults.^24^ Our study is the first to formally demonstrate the potential role of systemic inflammation in mediating the relationships between birth weight and AMI.

Finally, although birth weight is the exposure of interest in our analysis, birth weight is a proxy indicator for intrauterine exposures that affect growth. That is, birth weight is a downstream manifestation of other ‘root’ exposures (eg, genetics, maternal pregnancy exposures). Evaluation of such factors may provide new perspectives into the underlying causal mechanisms of the associations observed between birth weight and cardiovascular health.

### Associations with CMR metrics

Previous studies of the association between birth weight and cardiovascular imaging phenotypes are limited to preterm birth or SGA individuals. There is heterogeneity within and across these study samples with multiple factors potentially influencing birth weight and subsequent cardiovascular risk. As such, direct comparison across cohorts is not possible.

In an analysis of the Cardiovascular Risk in Young Finns Study participants, Arnott *et al* 25 compared echocardiography findings of 157 SGA adults with 627 individuals born average size for gestational age, reporting larger LV volumes and lower LVSV in the preterm cohort. Lewandowski *et al* 26 present a CMR study of 102 adults born preterm and 132 born at term. They reported greater LV mass, smaller internal diameters and poorer LV strain metrics in those with a history of preterm birth compared with those born at term. In a similar study, Goss *et al* 27 compare CMR metrics in 58 adolescents and adults born preterm with those of 52 age-matched participants born at term; the preterm cohort had significantly lower LV volumes compared with matched controls, as in the study by Lewandowski *et al*.^26^ These observations are corroborated by Mohamed *et al* 28 who similarly report higher CMR-derived LV mass index, reduced LV function and smaller LV volumes among 200 preterm individuals compared with 268 individuals born at term. The reduced volumes in preterm-born adults have been shown to relate to an impaired myocardial functional reserve,^29^ with functional impairments related to an increase in diffuse myocardial fibrosis.^30^

In our study of 19 314 population-based participants, we demonstrate a similar pattern of associations of lower birth weight with adverse CMR phenotypes. Specifically, we note association of lower birth weight with greater concentric LV remodelling and lower LVSV. These findings suggest that the adverse cardiovascular remodelling patterns related to lower birth weight are not limited to individuals with a history of preterm birth or SGA, but rather extend to a much larger proportion of the general population who have lower birth weight outside of these specific contexts.

### Limitations

The birth weight variable was based on self-report and may be subject to recall bias, which may add noise or bias to observed associations. Some of the other covariates were also ascertained from self-report. Misclassification in these variables may be non-differential with individuals with higher education and health status more likely to accurately recall their birth weight and parental medical history. The unavailability of gestational age precludes definitive distinction between preterm, SGA and small but term births. We were also unable to consider the impact of adverse pregnancy outcomes, such as pre-eclampsia or gestational diabetes. We were underpowered to study ethnicities other than white. There is an interval of several years between baseline recruitment and the imaging visit, which may have led to survival bias in the associations with CMR metrics. The nature of the study precludes causal inference, and we cannot exclude residual confounding.

## Conclusions

In this large population-based cohort of middle-aged adults, lower birth weight was associated with significantly increased risk of incident MI below a threshold of 3.2 kg (approximating population average birth weight), independent of socioeconomic, parental and childhood factors. Poor glycaemic control, adverse lipid profile, hypertension and systemic inflammation were significant independent mediators of this relationship. However, these factors explained less than one-third of the birth weight–AMI effect, indicating important influence of other biological pathways. Lower birth weight was linked to an unhealthy pattern of cardiovascular remodelling indicating greater concentricity and poorer LV function.

Our findings indicate that the adverse cardiovascular effects of low birth weight described in preterm/SGA cohorts may extend significantly into the general population. Further research is required to determine whether inclusion of birth weight may improve risk stratification and if preventative strategies targeted at individuals with lower birth weight have a role in improving clinical outcomes.

## Data Availability

Data may be obtained from a third party and are not publicly available. This research was conducted using the UK Biobank resource under access application 2964. UK Biobank will make the data available to all bona fide researchers for all types of health-related research that is in the public interest, without preferential or exclusive access for any persons. All researchers will be subject to the same application process and approval criteria as specified by UK Biobank. For more details on the access procedure, see the UK Biobank website: http://www.ukbiobank.ac.uk/register-apply.
