# Supplementary material for: Lower birth weight is linked to poorer cardiovascular health in middle-aged population-based adults
Source: Heart. 2022 Nov 16;109(7):535–41. doi: 10.1136/heartjnl-2022-321733 (PMC10086465; doi:10.1136/heartjnl-2022-321733)
Supplement: Supplementary data [file heartjnl-2022-321733supp001.pdf]

Supplementary Figure 1. Directed acyclic graph sets out the postulated causal relationships between covariates relevant to structure of our models

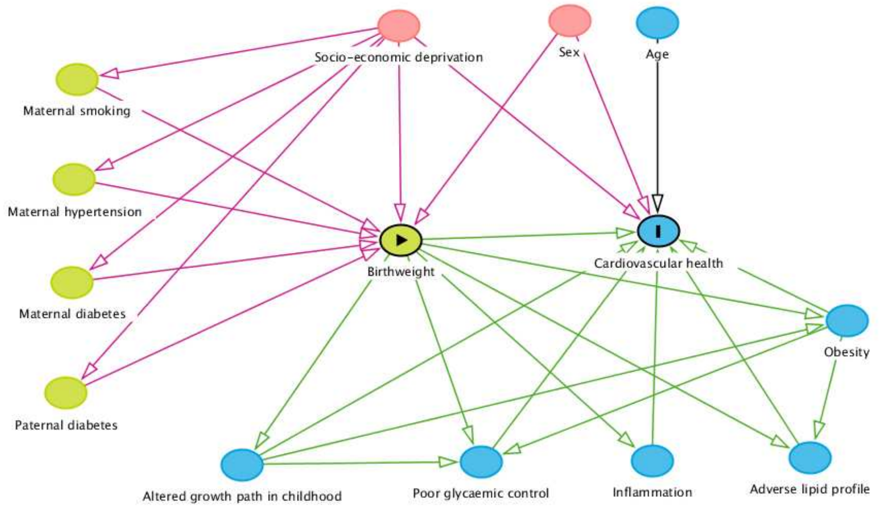

Supplementary Figure 1 footnote. Figure created using the online DAGitty tool (Johannes Textor, Benito van der Zander, Mark K. Gilthorpe, Maciej Liskiewicz, George T.H. Ellison. Robust causal inference using directed acyclic graphs: the R package 'dagitty'. *International Journal of Epidemiology* 45(6):1887-1894, 2016.)

Supplementary Figure 2. Flow chart of participants included in the study

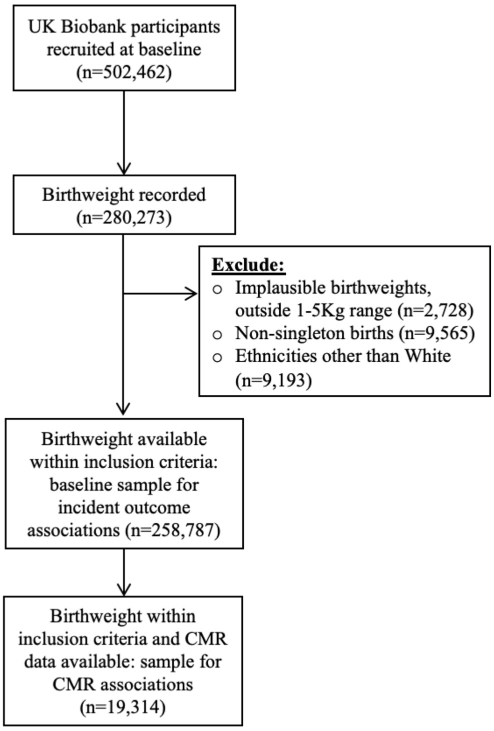

Supplementary Figure 2 footnote. CMR: cardiovascular magnetic resonance.

Supplementary Table 1. Ascertainment of covariates and degree of missingness

Confound/Mediator	UK Biobank field	Description	Data type	Missingness (n) %
Age	21003	Calculated from date of birth and date of baseline visit (recruitment)	continuous	0
Sex	31	Acquired from central registry at baseline (mix of NHS recorded and self-report)	Binary	0
Deprivation	189	Townsend deprivation index*, was calculated immediately prior to participant joining UK Biobank, based on the preceding national census output areas. Each participant is assigned a score corresponding to the output area in which their postcode was located.	Continuous	338 (0.1%)
Maternal diabetes	20110	Self-report at baseline	Binary	0
Paternal diabetes	20107	Self-report at baseline	Binary	0
Maternal hypertension	20110	Self-report at baseline	Binary	0
Maternal smoking	1787	Self-report at baseline	Binary	0
Diabetes	20002<br>6117<br>6153<br>2443	Self-report of diagnosis of diabetes or medications used for diabetes across several touchscreen and nurse interview questions	Binary	0
High cholesterol	20002<br>6117<br>6153	Self-report of diagnosis of high cholesterol or medications used for high cholesterol across several touchscreen and nurse interview questions	Binary	0
Hypertension	20002<br>6117<br>6153	Self-report of diagnosis of hypertension or medications used for hypertension across several touchscreen and nurse interview questions	Binary	0
Obesity	21001	Body mass index calculated from physical measurement of height and weight at baseline visit	Continuous	811 (0.3%)
Childhood height	1697	Self-report of comparative height at age 10	Ordinal	2802 (1.0%)
Childhood weight	1687	Self-report of comparative weight aged 10 years	Ordinal	2813 (1.1%)
Glycated haemoglobin	30750	Blood biochemistry at baseline**	Continuous	17298 (6.7%)
Glucose	30740	Serum biochemistry measurement	Continuous	37141 (14.4%)
Low density lipoprotein	30780	Serum biochemistry measurement	Continuous	16724 (6.5%)
High density lipoprotein	30760	Serum biochemistry measurement	Continuous	36996 (14.3%)
Triglyceride	30870	Serum biochemistry measurement	Continuous	16454 (6.4%)
Apolipoprotein A	30630	Serum biochemistry measurement	Continuous	65160 (25.2%)
C reactive protein	30710	Serum biochemistry measurement	Continuous	16778 (6.5%)

Supplementary Table 1 footnote. NHS: national health service. *Townsend, P., Phillimore, P., & Beattie, A. (1988). Health and Deprivation: Inequality and the North. *Nursing Standard*, *2*(17), 34–34. https://doi.org/10.7748/ns.2.17.34.s66. **The UK Biobank blood biochemistry companion document details assays and protocols for biochemistry measurements: https://biobank.ndph.ox.ac.uk/showcase/ukb/docs/serum_biochemistry.pdf.

Supplementary Table 2. Observed outcomes during the follow-up period*

Outcomes	Absolute number of outcomes	Rate of outcomes per 1000 patient years
All-cause mortality	8,354	3.62
CVD mortality	1,603	0.70
IHD mortality	845	0.37
Fatal AMI	325	0.14
Incident AMI	3,637	1.76

Supplementary Table 2 footnote. AMI: acute myocardial infarction; CVD: cardiovascular disease; IHD: ischaemic heart disease. *Total person time was 2,304,749 years (median 9.0 years [8.3 to 9.7]) for mortality outcomes, and 2,069,086 years (median 8.2 years [7.5 to 8.8]) for incident AMI.

Supplementary Table 3. Test for non-linearity of birthweight effect on outcomes

	p-value non-linear* vs. linear model
All-cause mortality	0.131
CVD death	0.024
IHD death	0.089
Fatal AMI	0.299
Incident AMI	0.006

Supplementary Table 3 footnote. *Cubic spline model with number of knots selected by AIC in the fully adjusted model. significance level $p<0.01$. AMI: acute myocardial infarction, CVD: cardiovascular disease, IHD: ischaemic heart disease

Supplementary Table 4. Comparison of model fit for incident AMI using cubic spline models with three, four, and five knots

	AIC
Linear	87079.32
Cubic spline – 3 knots	87073.89
Cubic spline – 4 knots	87076.08
Cubic spline – 5 knots	87078.05

Supplementary Table 4 footnote. AIC: Akaike information criterion, AMI: acute myocardial infarction

Supplementary Table 5. The independent effect of birthweight on incident AMI through individual mediators expressed as hazard ratios and Percentages of effect mediated (complete case analysis)

Mediators	Indirect effect of birthweight B(se)	Proportion of effect mediated
Comparative weight aged 10 years	0.998 (0.99 to 1.006)	-1.17%
Comparative height aged 10 years	1.009 (0.998 to 1.021)	5.84%
BMI	1.002 (0.993 to 1.004)	1.23%
Diabetes[$]	1.002 (1.0004 to 1.004)	1.30%
High cholesterol*[$]	1.006 (1.006 to 1.011)	4.09%
Hba1c*	1.011 (1.007 to 1.014)	6.99%
Glucose	0.998 (0.965 to 0.999)	-1.30%
LDL	1.003 (1.000 to 1.006)	1.88%
HDL*	1.008 (1.005 to 1.116)	5.19%
Lipoprotein a	1.000 (0.999 to 1.001)	-0.05%
Triglyceride level	1.004 (1.000 to 1.008)	2.40%
CRP*	1.010 (1.007 to 1.013)	6.36%
Hypertension*[$]	1.013 (1.010 to 1.017)	8.44%
Total proportion mediated through all mediators	–	41.23%
Total proportion mediated through significant mediators	–	31.08%

Supplementary Table 5 footnote: There was no evidence of multicollinearity by VIF <10. *significant variables with Significance level set at p-value <0.004 correcting for 13 mediators. Total effect (95% CI): 1.17 (1.07-1.27). Direct effect (95% CI): 1.11 (1.02-1.21). [$]The effects shown are conditional effects for a male individual with average age and Townsend score and without maternal or paternal risk factors.

Supplementary Table 6. The independent effect of birthweight on incident AMI through individual mediators expressed as hazard ratios and Percentages of effect mediated (with and without imputed variables)

Mediators	Proportion of effect mediated (no imputation)	Proportion of effect mediated (after imputation)
Comparative weight aged 10 years	-1.17%	-0.68%
Comparative height aged 10 years	5.84%	5.12%
BMI	1.23%	0.96%
Diabetes	1.30%	1.36%
Hypercholesterolaemia	4.09%	4.78%
Hba1c	6.99%	6.48%
Glucose*	-1.30%	-1.16%
LDL	1.88%	0.96%
HDL *	5.19%	4.57%
Lipoprotein A*	-0.05%	-0.07%
Triglyceride level*	2.40%	1.71%
CRP*	6.36%	6.96%
Hypertension	8.44%	10.23%
Total proportion mediated through all mediators	41.23%	41.20%
Total proportion mediated through significant mediators	31.08%	34.38%

Supplementary Table 6 footnote: There was no evidence of multicollinearity by VIF <10.
*significant variables with significance level set at p-value <0.004 correcting for 13 mediators.
